# Perspectives on Matrix Metalloproteinase-8 and Salivary Osteoprotegerin in Orthodontic Strategy in Children with Chronic Kidney Disease

**DOI:** 10.3390/jcm14092951

**Published:** 2025-04-24

**Authors:** Natalia Sergeevna Morozova, Alina Alekseevna Elovskaya, Ekaterina Andreevna Maslikova, Andrey Vladimirovich Sevbitov, Maria Dmitrievna Timoshina, Malkan Abdrashidovna Amkhadova, Larisa Dmitrievna Maltseva, Ellina Valerievna Velichko, Elena Yur’evna Danilova, Olga Leonidovna Morozova

**Affiliations:** 1 Department of Dental Diseases Propaedeutics, E.V. Borovsky Institute of Dentistry, I.M. Sechenov First Moscow State Medical University (Sechenov University), 117418 Moscow, Russiasevbitov_a_v@staff.sechenov.ru (A.V.S.); timoshina_m_d@staff.sechenov.ru (M.D.T.); 2 Department of Pediatric, Preventive Dentistry and Orthodontics, E.V. Borovsky Institute of Dentistry, I.M. Sechenov First Moscow State Medical University (Sechenov University), 121059 Moscow, Russia; elovskaya_a_a@staff.sechenov.ru (A.A.E.); maslikova_e_a@staff.sechenov.ru (E.A.M.); 3 Department of Surgical Dentistry and Implantology of the Faculty of Advanced Medical Studies Moscow Regional Research and Clinical Institute (“MONIKI”), 129110 Moscow, Russia; amkhadova@mail.ru; 4 Department of Pathophysiology, Institute of Digital Biodesign and Modeling of Living System, I.M. Sechenov First Moscow State Medical University (Sechenov University), 119019 Moscow, Russia; maltseva_l_d@staff.sechenov.ru (L.D.M.); velichko_e_v@staff.sechenov.ru (E.V.V.); morozova_o_l@staff.sechenov.ru (O.L.M.); 5 Molecular Theranostics Institute, Biomedical Science and Technology Park, I.M. Sechenov First Moscow State Medical University (Sechenov University), 119991 Moscow, Russia; 6 Department of Chemistry, M.V. Lomonosov Moscow State University, 119991 Moscow, Russia

**Keywords:** biomarkers, salivary, bone mineral density, orthodontic pathology, chronic kidney disease (CKD), children

## Abstract

**Background/Objectives**: This study aimed to establish the regularities of changes in the content of matrix metalloproteinase 8 (MMP-8) and osteoprotegerin (OPG), the most well-known indicators of bone metabolism disorders, in the saliva of children with different severities of chronic kidney disease (CKD) who need orthodontic treatment. **Methods**: The study of MMP-8 and OPG content in saliva was carried out in 76 children in need of orthodontic treatment, who were divided into equal groups (G) of 19 people: G1—children with congenital malformations of the urinary tract, acquired renal pathology, and CKD stage 1 and 2, receiving medical therapy, as well as more having a deep distal bite formed by mandibular micrognathia; G2—children with a terminal stage of CKD, receiving renal replacement therapy in the volume of hemodialysis, with a characteristic distal bite of different etiology; G3—children one year after kidney transplantation, with a tendency to form an open distal bite, associated to a greater extent with maxillary macrognathia. G4—practically healthy children without renal pathology stratified by sex and age. **Results**: It was found that the content of MMP-8 and OPG in the saliva of children with different CKD stages who needed orthodontic treatment was significantly higher than the G4. The maximum values of MMP-8 were registered in G2. An increase in OPG content in saliva was observed in the G1 and G3. **Conclusions**: The identified changes in markers of mineral and bone disorders in the saliva of children with different stages of CKD show the possibility of their use as non-invasive predictive and prognostic markers for the diagnosis of preclinical stages of bone metabolic disorders.

## 1. Introduction

Chronic kidney disease (CKD) in children is accompanied by the development of systemic disorders of mineral and bone metabolism and changes in the dentoalveolar system (DAS) [1,2,3]. The development of mineral and bone disorders in pediatric patients with CKD leads to difficulties in dental treatment and adverse outcomes, especially in the terminal stage of CKD [4,5]. This patient cohort requires a personalized orthodontic treatment strategy.

In recent years, much attention has been paid to non-invasive methods of diagnosing various oral pathologies in children with kidney disease, including the development of dentoalveolar anomalies [6,7]. Nowadays, there is no single algorithm for preventing dental disorders in children with CKD and choosing an orthodontic treatment method that correlates with the state of the bone tissue in the maxillofacial region.

At the same time, poor oral hygiene can be a contraindication to orthodontic treatment with fixed appliances, as it will create a cariogenic situation in the oral cavity and provoke periodontal diseases. The use of removable and fixed orthodontic appliances is often complicated by traumatization of the oral mucosa by structural elements [8]. Trauma of the mucosa occurs against the background of pronounced xerostomia, which can lead to the accession of secondary infection and the development of more severe forms of oral mucosa diseases [9,10]. Various factors can cause the development of xerostomia in children with CKD: limited fluid intake, medication or mouth breathing. According to the results of the study by S.V. Chuikin et al. 84.6 ± 5% of children with CKD on programmed dialysis complain of dry oral mucosa, compared to the control group of children without chronic diseases, in whom complaints of xerostomia are observed only in 3.3 ± 3.3% of cases [11].

Several researchers have identified the need to assess bone mineral density in patients diagnosed with CKD, as this disease is often associated with decreased bone density, impaired mineralization, osteoporosis, and risk of bone fractures [12,13]. When bone density is insufficient, such as in osteoporosis, there is an increased risk of pathological resorption and complications such as excessive mobility due to the predominance of an osteoclast function [14]. Conversely, decreased bone density can delay or even make orthodontic tooth movement impossible [15]. Modern imaging techniques such as cone beam computed tomography (CBCT) provide detailed information on bone density and structure, revealing anatomical features that can influence treatment planning [16,17].

Data indicate the promising application of saliva as a biological fluid for determining various damage markers to the elements of the DAS [18,19,20]. In addition, saliva has the advantage of easy and non-invasive collection. It can be used as a gold standard for the early detection and monitoring of periodontitis in patients with CKD, which is especially important in managing children [19,20,21]. Matrix metalloproteinase 8 (MMP-8) [22,23,24,25] and osteoprotegerin (OPG) [26,27,28] are considered the best-known indicators of bone metabolic disorders.

MMP-8 plays an important role in the development of destruction of all peri-implant bed tissues and is expressed exclusively in inflammatory conditions, including the periodontium, which induces bone resorption [21]. The active form of MMP-8 cleaves fibronectin, cartilage aggrecan, serpins and peptides such as angiotensin and substance P, thereby participating in the degradation of the extracellular matrix [28]. MMP-8 serves as an informative biomarker reflecting the degree of periodontal inflammation [21]; its determination is essential in early, subclinical diagnosis of periodontitis for preventive treatment and prevention of tooth loss [23]. Progression of periodontitis is a significant risk factor for the development of osteonecrosis of the jaws in patients with CKD [24]. Monitoring the level of this biomarker at all stages of treatment, including surgical correction of oral pathology, will help to judge the effectiveness of therapy [25].

OPG, an osteoclastogenesis inhibitory factor, belongs to the tumor necrosis factor receptor superfamily and is an essential link in the RANK (receptor activator of nuclear factor kappa-B)/RANKL (receptor activator of nuclear factor kappa-B ligand) OPG system. By binding to RANK instead of RANKL on osteoclasts, OPG prevents bone resorption [26]. Mechanical unloading or decreased mechanical stimulation can increase the mRNA expression of OPG and RANKL [29] and the balance between these two molecules predetermines normal bone mineral density [25]. The analysis of this marker in saliva is significant in terms of periodontal treatment outcomes and prognosis of bone changes in the DAS. It is known that with timely treatment of periodontitis, the initially elevated level of OPG decreased [27]. 

Thus, MMP-8 and OPG are promising for diagnosing the early stages of bone metabolism disorders in children with CKD and can be considered predictive and prognostic markers. The analysis of correlations between biomarkers in saliva, dental indices, and the density of bone structures in jaw bones allowed us to develop a personalized strategy of orthodontic care.

The study aimed to establish patterns of changes in the content of MMP-8 and OPG in the saliva of children with different severities of CKD.

## 2. Materials and Methods

### 2.1. Subjects

The clinical stage was carried out from September 2023 to May 2024 in the State Budgetary Institution ‘Morozovsky Children’s City Clinical Hospital of the Moscow City Health Care Department’, the Federal State Autonomous Institution of the Ministry of Health of the Russian Federation ‘National Medical Research Center for Children’s Health’, the State Budgetary Institution ‘G.N. Speransky Children’s City Clinical Hospital No. 9 named after G.N. Speransky’ of the Moscow City Health Care Department’, the Federal State Budgetary Institution of the Ministry of Health of the Russian Federation ‘National Medical Research Center of Transplantology and Artificial Organs named after V. I. Shumakov’, the E.V. Borovsky Institute of Dentistry of the I.M. Sechenov First Moscow State Medical University (Sechenov University). The study was approved by the local ethical committee of the I.M. Sechenov First Moscow State Medical University (Sechenov University). Extract from the protocol No. 01-22 from 20 January 2022.

The study was conducted on 76 children from 7 to 18 years old (mean age 11.4 ± 3.8). Of these, 57 were children with various stages of CKD and a background of congenital and acquired kidney diseases with orthodontic pathology including skeletal and dental-alveolar forms of distal, open and deep bite, accompanied by various degrees of severity of macro- and micrognathia of the upper and lower jaws, as well as pathological inclination of the frontal group of teeth (protrusion, retrusion). The control group included 19 practically healthy children with the same orthodontic pathology.

Exclusion criteria were age younger than 7 and older than 18 years, intercurrent forms of infectious and inflammatory diseases, sepsis, concomitant pathology (diabetes mellitus, respiratory failure, cardiovascular failure), lack of parental/representative consent for their children to participate in the study, previous orthodontic treatment, previous surgery or trauma to the maxillofacial region.

**Figure 1:**
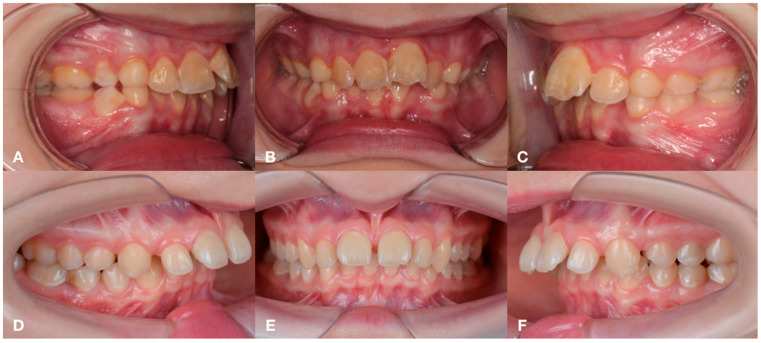
The intraoral photo protocol of the patients in Group 1 with a deep distal bite formed by mandibular micrognathia. Patient 1: occlusion on the right (**A**); occlusion in front (**B**); occlusion on the left (**C**). Patient 2: occlusion on the right (**D**); occlusion in front (**E**); occlusion on the left (**F**).

**Figure 2:**
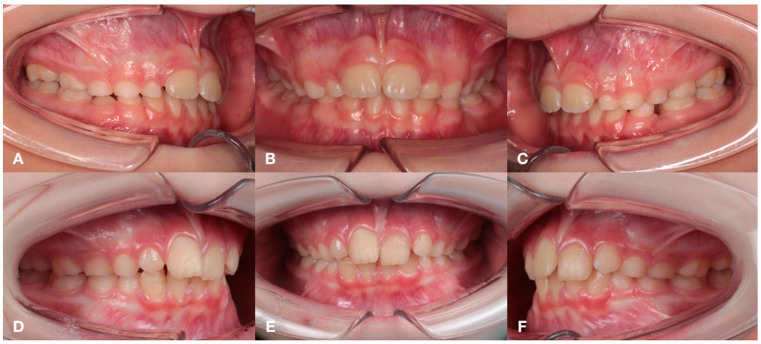
The intraoral photo protocol of the patients in Group 2 with a distal bite of different etiology. Patient 1: occlusion on the right (**A**); occlusion in front (**B**); occlusion on the left (**C**). Patient 2: occlusion on the right (**D**); occlusion in front (**E**); occlusion on the left (**F**).

**Figure 3:**
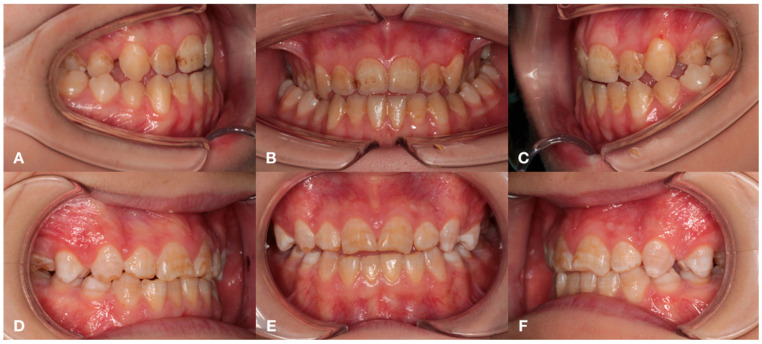
The intraoral photo protocol of the patients in Group 3 with an open distal bite, associated to a greater extent with maxillary macrognathia. Patient 1: occlusion on the right (**A**); occlusion in front (**B**); occlusion on the left (**C**). Patient 2: occlusion on the right (**D**); occlusion in front (**E**); occlusion on the left (**F**).

**Figure 4:**
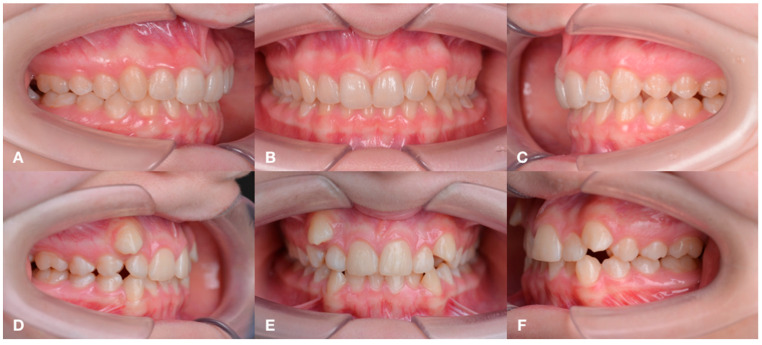
The intraoral photo protocol of the patients in Group 4 having different orthodontic abnormalities. Patient 1: occlusion on the right (**A**); occlusion in front (**B**); occlusion on the left (**C**). Patient 2: occlusion on the right (**D**); occlusion in front (**E**); occlusion on the left (**F**).

Depending on the stage of CKD and treatment, the following groups were formed: Group 1 (*n* = 19)—children with congenital malformations of the urinary tract, acquired renal pathology and CKD stage 1 and 2, receiving medical therapy, as well as more having a deep distal bite formed by mandibular micrognathia (Figure 1); group 2 (*n* = 19)—children with a terminal stage of CKD, receiving renal replacement therapy (RRT) in the volume of hemodialysis, with a characteristic distal bite of different etiology (Figure 2); group 3 (*n* = 19) — children one year after kidney transplantation, with a tendency to form an open distal bite, associated to a greater extent with maxillary macrognathia (Figure 3); group 4—practically healthy children, without renal pathology, stratified by sex and age (Figure 4). The following images verify general differences in the intraoral photo protocol of the chosen groups.

**Figure 5:**
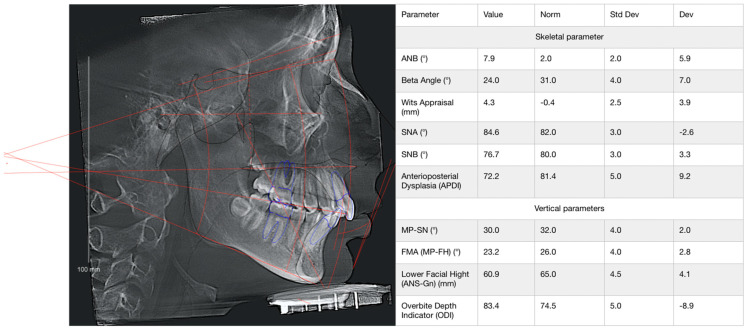
The 3D cephalometric analysis of the patient from group 1 demonstrates a distal bite (reduction in Betta angle and ANB angle) and a deep bite (reduction in MP-SN angle).

**Figure 6:**
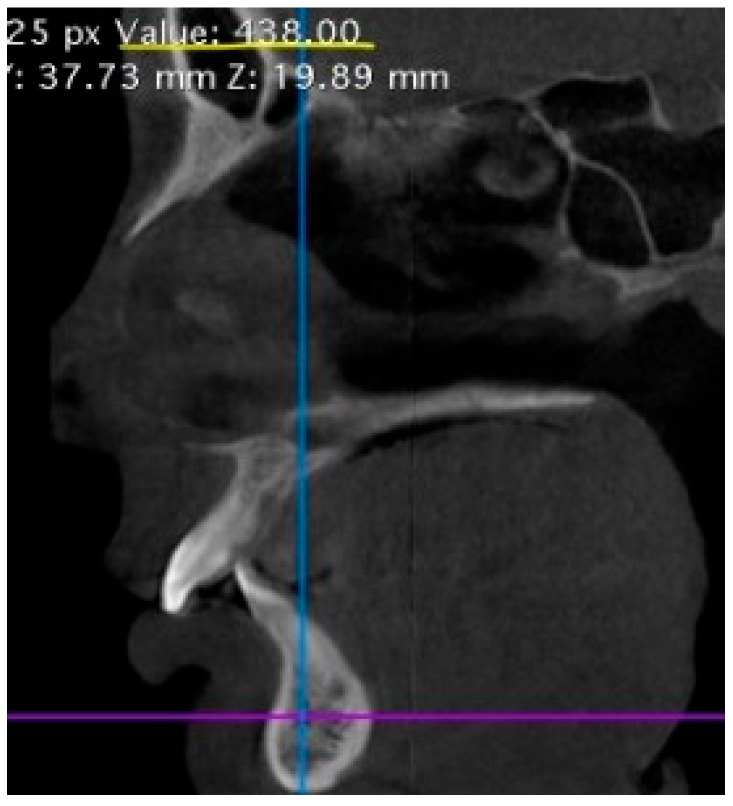
A radiological density of the mandibular alveolar process in the region of the tooth 4.2 = 438 HU, which is characterized as porous cortical and thin trabecular bone (D3) according to the C. Mish classification.

Dental status was assessed based on external and intraoral examinations. Dental anomalies were evaluated based on photometric analysis (intraoral and extraoral photo protocol), clinical and instrumental analysis of plaster models of the jaws, and cephalometric analysis of the skull based on cone beam computed tomography (CBCT) data (Figure 5). The prevalence of inflammatory and non-inflammatory changes in oral soft tissues was considered. The following indices were used to assess the state of periodontal tissues: periodontal gingival sulcus bleeding index (SBI), papillary marginal alveolar (PMA) index and Russell index. The assessment of radiological bone density was based on the mathematical reconstruction of X-ray attenuation coefficients assigned to each pixel for image display and expressed in Hounsfield units (HUs) according to the classification of S. Mish (Figure 6).

### 2.2. Saliva Samples Analysis

The content of MMP-8 and OPG in saliva was determined in all patients. Saliva was collected 1 h before meals in the morning. The whole saliva was collected by an absorptive method using a sterile dental cotton swab. After the swab was soaked with saliva, it was placed in a sterile Falcon tube, and the saliva was separated from the swab by centrifugation. Then, the biomaterial samples were transferred into Eppendorf tubes supplemented with 0.2% ProClin™ 300 biocidal solution. The tubes were shaken using a centrifuge-vortex Elmi CM-70M-07 for 10 min and stored at −80 °C until the beginning of the study. MMP-8 and OPG were analyzed by a solid-phase enzyme-linked immunosorbent assay (ELISA) using reagent kits: HumanTotal MMP-8—Quantikine^®^, R&DSystems, Minneapolis, MN, USA; and Osteoprotegerin, Biomerica, Irvine, CA, USA.

### 2.3. Statistical Analysis

Before hypothesis testing, the Shapiro–Wilk test was used to assess data distribution normality, and the Levene’s test examined homogeneity of variance. Continuous variables are presented as mean ± SD for variables with parametric distribution or median (25th–75th percentile) for non-parametric variables. Comparisons between groups were performed using one-way analysis of variance (ANOVA) and a Holm–Sidak test for multiple comparisons or Kruskal–Wallis and Dunn’s tests for multiple comparisons, when appropriate. Sample size calculations ensured 80% statistical power at α = 0.05. A Spearman’s rank correlation test was used to analyze the correlation between Haunsfield indexes and biochemical variables. A *p*-value of less than 0.05 (two-sided) was considered statistically significant. The statistical software packages GraphPad Prism Software 8.0.1 (GraphPad, Boston, MA, USA) and MetaboAnalyst 6.0 were used for statistical analyses. Data were entered and organized using Microsoft Office Excel 2013. An a priori power analysis (one way ANOVA, effect size f = 0.40, α = 0.05, power = 0.80) performed with G*Power 3.1 indicated a minimum of 15 participants per group; therefore, 19 were enrolled in each group to accommodate potential dropouts.

## 3. Results

### 3.1. Periodontal Status and Inflammatory Indices

The results of the periodontal tissue assessment, which were evaluated by SBI, the periodontal inflammation index PMA, and the Russell index, are presented in Table 1.

In group 1 of children with CKD stage 1 and 2 receiving medical therapy, the indices of prevalence and intensity of periodontal inflammatory diseases and SBI did not differ from those of group 4 (control) (Table 1). When assessing the severity and prevalence of inflammatory changes using the PMA index, the average degree of inflammation of periodontal tissues was found in groups 2 and 3, and the mild degree of gingival inflammation was found in groups 1 and 4. The highest value of the PMA index was observed in the group of children with terminal-stage CKD receiving renal replacement therapy in the volume of hemodialysis. In the group of children after kidney transplantation, the index slightly decreased. When assessing the Russell periodontal index, an initial and mild degree of periodontal pathology was registered in all examined children. The highest index value was found in group 2 children with terminal CKD. A decrease in the index was observed in children of group 3.

### 3.2. Radiological Bone Density of the Jaws, Hounsfield Index Analysis

When analyzing the radiological bone density of the jaws according to the Hounsfield index, a decrease in HU in the anterior and posterior regions of the mandible and the posterior region of the maxilla was found in all the studied groups compared to the control group (Table 2). In the anterior maxilla, a decrease in HU was noted in the groups with renal replacement therapy in the volume of dialysis and after transplantation to the control group.

In particular, children with terminal stage CKD (group 2) demonstrated the lowest HU values in both the mandible and maxilla, comparable only in level to the anterior maxilla data of group 3. This finding suggests a more advanced reduction in bone density compared to stage 1–2 CKD (group 1). At the same time, HU values in group 3 were intermediate, generally higher than in group 2, but still below control values. The anterior part of the mandible displayed the most significant variations in bone density between these groups due to its potential susceptibility to kidney disease-related bone changes. The posterior mandible and posterior maxilla were significantly affected, consistent with decreased jawbone density in CKD.

### 3.3. Matrix Metalloproteinase-8 and Osteoprotegerin Levels in Saliva in Comparison to eGFR

**Figure 7:**
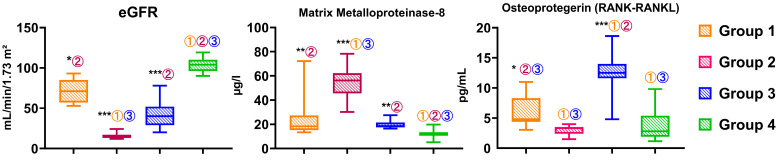
The picture shows the estimated glomerular filtration rate (eGFR) and salivary markers (matrix metalloproteinase-8 (MMP-8) and osteoprotegerin (OPG)) in the four groups that were studied. The asterisks indicate that there are statistically significant differences from the control group *p*-value < 0.05 (*), <0.005 (**), and <0.0005 (***). The circled numbers show significant differences (*p* < 0.05) compared to Group 1 (①), Group 2 (②), and Group 3 (③).

Two biochemical parameters, matrix metalloproteinase-8 (MMP-8) and osteoprotegerin (OPG) concentrations, were assessed in four groups of participants differing in renal function status (see Figure 7). The figure also presents values of the estimated glomerular filtration rate (eGFR).

The MMP-8 level was significantly increased in all studied groups compared to the control (*p* < 0.005; *p* < 0.0005; *p* < 0.005, respectively) (Figure 7). At the same time, its maximum values were registered in the group 2 children on hemodialysis replacement therapy (*p* < 0.05). The broadest range of values was observed in patients from Groups 1 and 2, indicating significant interindividual variability in the degree of inflammation or tissue remodeling. Consequently, the progression of CKD to its terminal stage is accompanied by the most significant increase in MMP-8. In contrast, the partially preserved or restored renal function (groups 1 and 3) is associated with a moderate increase in this marker relative to normal. The results are consistent with the clinical data of periodontal tissues and indicate the severity of lesions of both hard and soft tissues of the oral cavity in children with the terminal stage of CKD.

In group 3, median OPG values were statistically significantly higher than in all other groups included in the study (Figure 7). While OPG values in patients in the initial stages of CKD (group 1) were lower than in group 3, they were also higher than in the control and the terminal stage groups. The marker values in the control group of patients were positioned intermediately between groups 1 and 2: Osteoprotegerin levels were markedly lower than in patients in the initial CKD stages but higher than in patients in the terminal stage. The latter (group 2) were characterized by the lowest OPG values (median 3.3) and the narrowest range of values among the studied samples, repeating the general pattern of eGFR data and anterior and posterior jaw density values according to CBCT data. Therefore, a correlation analysis of the features studied was performed.

### 3.4. Correlation Analysis of Renal Function, Bone Density, and Salivary Biomarkers

**Figure 8:**
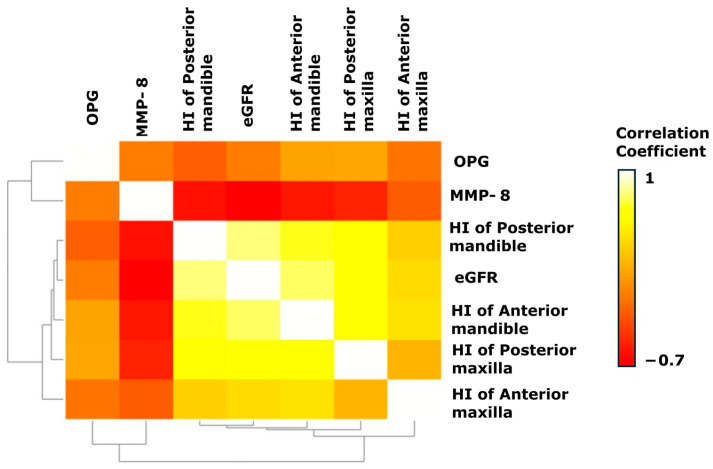
Heatmap of Spearman’s rank correlation coefficients (r_s_) for all studied features across the four groups. The color intensity reflects the strength and direction of the correlation, with the scale optimized from +1.0 to −0.7 based on the observed data.

The correlations between all parameters were evaluated by a Spearman’s rank correlation coefficient test. All subjects were included in the analysis. The heatmap provides visualization of discovered variable relationships (Figure 8).

The heatmap demonstrates a positive correlation pattern between eGFR and HU measurements in both anterior and posterior jaw regions, which is consistent with the literature. The inverse correlation between MMP-8 and eGFR, along with its negative association with HU values in both the mandible and maxilla, were also statistically significant at *p* < 0.05. The correlation patterns for OPG showed lower strength while maintaining fewer statistically substantial findings with bone density or eGFR.

To conclude, the heatmap pattern representing improved renal function (eGFR) creates a linked connection that leads to an increase in jaw density, and high levels of MMP-8 indicate lower skeletal parameters in the body.

## 4. Discussion

The results of periodontal tissue assessment by a periodontal inflammation index PMA and Russell index confirmed the most significant changes in children of group 2 with terminal CKD and in group 3 after kidney transplantation. Analysis of the content of mineral and bone changes markers in saliva showed that the levels of MMP-8 and OPG were significantly increased in all studied groups compared to the control. The maximum values of MMP-8 were registered in children of group 2 with CKD on hemodialysis and OPG in group 3 after kidney transplantation.

The number of patients with DAS lesions in the setting of CKD continues to increase progressively [30,31]. A special category is children with terminal CKD who are on hemodialysis [4,32,33]. It should be noted that such changes in oral tissues as gingival hyperplasia, enamel hypoplasia, petechiae, and bleeding from the gums are risk factors for reducing the quality of life in children with CKD but lead to seeking dental care [1,4,34]. Diagnosis of damage to the soft tissues of the oral cavity is limited to examination, and hard tissues are limited to the determination of serum parathormone levels and radiological methods, which are informative only at the extreme bone changes [2,35]. On the one hand, dentists deal with advanced variants of the DAS pathology that require reconstructive surgeries and combined treatment methods, including preliminary surgical manipulations of temporary cortical support and mechanical devices [35]. On the other hand, pronounced mineral disorders in children with CKD cause a high risk of jawbone fracture even during tooth extraction, which in some cases does not allow the necessary treatment complex to be carried out in full. As a rule, the development of changes in bone tissue in children correlates with the progression of CKD, which is associated with impaired parathyroid hormone production, hypocalcaemia, hypophosphatemia, and metabolic acidosis [3,36,37]. In a study of bone and mineral metabolism in children with different causes of CKD development, it was shown that the most pronounced and severe changes in bone metabolism are found in children with nephropathic cystinosis, and the severity of skeletal comorbidity correlates with the stage of CKD [36]. The authors found that despite medical therapy of the Fanconi syndrome, the risk of short stature and bone deformity in these patients is 11 times higher than in children with other causes of CKD. This reflects the more severe impairment of mineralization processes and a more intense course of bone resorption that persists even after kidney transplantation in this group of patients [36]. Other researchers have also shown that skeletal complications in patients with cystinosis-associated metabolic bone disease are only partially correctable after kidney transplantation, and their severity in children is five times less than in adults. Nevertheless, they also correlate with the stage of CKD [3]. Dissatisfaction with the results of early diagnosis and treatment prompts the search for methods of timely diagnosis and the development of low-traumatic technologies for the correction of dental problems [6,20,21].

High values of MMP-8 in children of group 2 with terminal CKD on hemodialysis could be associated with a mild degree of periodontitis detected by changes in periodontal indices, as well as with the beginning of osteodystrophic changes in hard tissues of the oral cavity. In children of group 3 after kidney transplantation, the detected changes in biomarkers indicated both a mild course of periodontitis and the development of severe osteodystrophy in the form of fluorosis, enamel hyperplasia and its pathological abrasion. MMP-8 is a marker of not only destructive changes in bone tissue but also the severity of the inflammatory process in the oral cavity. The level of this factor in saliva can be used to judge the severity of periodontitis [22]. Given that the initial manifestations of this disease (gingivitis) are reversible, and its progressive course leads to tooth loss, subclinical diagnosis of periodontitis is important, especially for children receiving renal replacement therapy, both in the volume of hemodialysis and transplantation. Monitoring this factor’s level at all treatment stages, including surgical correction of oral pathology, will help judge the effectiveness of therapy [22,23]. This correlates with the data of other authors. Thus, in the work of researchers who studied the relationship between chronic periodontal diseases and the level of proinflammatory cytokines, including MMP-8, in patients on maintenance hemodialysis, it was shown that their levels of gingival fluid positively correlate with serum levels and with the clinical parameters of periodontal disease (*p* < 0.05) [19]. Other researchers have shown that it is active MMP-8 from gingival fluid that can be used as an additional, independent, and prophylactic biomarker for detection, monitoring of the course and effect of treatment, as well as prognosis of periodontal diseases in a noninvasive and convenient way for patients without causing bacteraemia, which is very important for patients with CKD [20].

Group 3 patients had lower hygiene and periodontal indices than group 2 children, which agrees with the results of Oduncuoğlu B.F. et al. [5] and requires professional oral hygiene at the earliest opportunity [38]. This is probably due to high doses of immunosuppressive drugs used to prevent kidney transplant rejection, demonstrating a less pronounced clinical picture of the manifestation of inflammatory changes in the oral cavity. In children after kidney transplantation, an increase in the level of OPG was revealed, which demonstrates a pronounced osteodystrophy, which is confirmed by clinical and radiological changes in bone density of the anterior and posterior parts of the mandible according to CBCT data. The results obtained regarding this biomarker have been confirmed by other studies, according to which the increased OPG level indicated a marked decrease in bone mineral density of the DAS bone tissue and a high risk of jaw fracture [38].

Our study has some limitations due to the lack of a comprehensive assessment of bone mineral changes in children with CKD, which requires invasive blood sampling and the determination of a wide range of parameters in serum: P content, albumin-adjusted total Ca, ionized Ca^2+^, parathormone, and bone fraction of alkaline phosphatase. The number and age of patients are limited by their admission to a specialized children’s hospital within the time frame of the study and by the diagnostic cut-off at a single follow-up point, as well as by the planning of the orthodontic treatment with mechanical-type appliances, which are indicated for use in children from the period of dentition change. Additional longitudinal research involving probabilistic participant selection throughout the disease progression is necessary to establish definitive conclusions about these markers.

## 5. Conclusions

The results of our study show the possibility of using MMP-8 and OPG in saliva to detect the initial stages of the development of periodontal inflammatory changes and bone mineral disorders in children with different stages of CKD. Diagnosis of minimal changes in the DAS in patients with CKD stages 1 and 2 provides timely and qualitative sanation of the odontogenic foci of infection. Determination of biomarkers in saliva expands the possibilities of non-invasive diagnostics of DAS diseases in children with CKD and is available for wide application in clinical practice. Further research in this area will allow the use of biomarkers to select a personalized orthodontic treatment strategy.

## Figures and Tables

**Table 1:** Evaluation of periodontal tissues in children with CKD. ^1^—*p* < 0.05 when comparing to 1 group; ^2^—*p* < 0.05 when comparing to 2 group; ^3^—*p* < 0.05 when comparing to 3 group, ^4^—*p* < 0.05 when comparing to 4 group. For normally distributed values, the values are given in Mean ± SD format.

Index	Group 1 (*n* = 19)	Group 2 (*n* = 19)	Group 3 (*n* = 19)	Group 4 (*n* = 19)
SBI	1.7 ± 0.51 ^2,3^	2.78 ± 0.61 ^1,3,4^	2.2 ± 0.49 ^1,4^	1.6 ± 0.43 ^2,3^
PMA	20 ± 4.57 ^2,3^	49 ± 11.14 ^1,4^	32 ± 7.63 ^1,2,4^	16 ± 3.95 ^2,3^
Russell	0.73 ± 0.09 ^2^	1.35 ± 0.3 ^1,3,4^	0.91 ± 0.23 ^4,2^	0.67 ± 0.08 ^2,3^

**Table 2:** Bone density in the study groups according to CBCT data. ^1^—*p* < 0.05 when comparing to 1 group; ^2^—*p* < 0.05 when comparing to 2 group; ^3^—*p* < 0.05 when comparing to 3 group, ^4^—*p* < 0.05 when comparing to 4 group. For normally distributed values, the values are given in Mean ± SD format, for values with non-normal distribution Median [Q1; Q3].

Sections of the Jaw	Group 1 (*n* = 19), HU	Group 2 (*n* = 19), HU	Group 3 (*n* = 19), HU	Group 4 (*n* = 19), HU
Anterior mandible	2790 [2200; 3010] ^1,3^	989.0 [940.0; 1122] ^1,3,4^	1749 [1570; 1999] ^1,2,4^	3096 [1980; 3554] ^2,3^
Posterior mandible	1560 [1200; 1770] ^2,3^	785.0 [690.0; 916.0] ^1,4^	850.0 [790.0; 940.0] ^1,4^	1800 [1384; 1960] ^2,3^
Anterior maxilla	534.5 ± 123.4	476.3 ± 71.3 ^4^	460.5 ± 66.2 ^4^	614.6 ± 171.7 ^2,3^
Posterior maxilla	378.9 ± 91.0 ^2^	207.9 ± 19.4 ^1,3,4^	302.5 ± 104.7 ^2,4^	432.5 ± 114.6 ^2,3^

## Data Availability

Data are unavailable due to privacy or ethical restrictions.

## References

[1] Andaloro C., Sessa C., Bua N., La Mantia I. (2018). Chronic kidney disease in children: Assessment of oral health status. Dent. Med. Probl..

[2] Sezer B., Kodaman Dokumacıgil N., Kaya R., Güven S., Türkkan Ö.N., Çiçek N., Alpay H., Kargül B. (2023). Association between serum biomarkers and oral health status in children with chronic kidney disease: A cross-sectional study. Clin. Oral Investig..

[3] Lahring J., Leifheit-Nestler M., Ewert A., Herzig N., Köppl C., Pott V., Oh J., Büscher A., Thumfart J., Weber L.T. (2024). Cystinosis-associated metabolic bone disease across ages and CKD stages 1-5D/T. J. Clin. Endocrinol. Metab..

[4] Velan E., Sheller B. (2021). Oral health in children with chronic kidney disease. Pediatr. Nephrol..

[5] Oduncuoğlu B.F., Alaaddinoğlu E.E., Çolak T., Akdur A., Haberal M. (2020). Effects of Renal Transplantation and Hemodialysis on Patient’s General Health Perception and Oral Health-Related Quality of Life: A Single-Center Cross-Sectional Study. Transplant. Proc..

[6] Vacaru R.P., Didilescu A.C., Constantinescu I., Mărunțelu I., Tănase M., Stanciu I.A., Kaman W.E., Brand H.S. (2022). Salivary Enzymatic Activity and Carious Experience in Children: A Cross-Sectional Study. Children.

[7] Keskin M., Rintamarttunen J., Gülçiçek E., Räisänen I.T., Gupta S., Tervahartiala T., Pätilä T., Sorsa T. (2023). A Comparative Analysis of Treatment-Related Changes in the Diagnostic Biomarker Active Metalloproteinase-8 Levels in Patients with Periodontitis. Diagnostics.

[8] Altaee Z.H., Bdaiwi A.T., Al-Salmany L.H.A., Khalaf B.Z. (2024). Changes in epithelial cells adjacent to orthodontic devices. Braz. Dent. Sci..

[9] Sharma T., Sharma A., Sharma A., Bansal C., Patyal A. (2022). Orthodontic management in medically compromised patients. Int. J. Sci. Rep..

[10] Hikmatuloyevna M.M., Komiljon o’g’li S.S., Komiljon o’g’li S.S. (2024). Oral mucosa injuries. Indones. J. Instr. Media Model..

[11] Morozova N.S., Elovskaya A.A. (2021). Manifestation of orofacial pathology in children with chronic kidney disease. Med. Pharm. J. Pulse.

[12] Shroff R., Wesseling-Perry K., Bacchetta J., Emma F., Goldstein S.L., Bagga A., Bates C.M., Shroff R. (2022). Chronic Kidney Disease–Mineral and Bone Disorder (CKD-MBD). Pediatr. Nephrol..

[13] Denburg M.R., Kumar J., Jemielita T., Brooks E.R., Skversky A., Portale A.A., Salusky I.B., Warady B.A., Furth S.L., Leonard M.B. (2016). Fracture Burden and Risk Factors in Childhood CKD: Results from the CKiD Cohort Study. J. Am. Soc. Nephrol..

[14] Nara Y. (2021). Analysis of Bone Microstructure and Orthodontic Tooth Movement in Osteoporosis Model Mice. Ph.D. Thesis.

[15] Li Y., Jacox L.A., Little S.H., Ko C.C. (2018). Orthodontic tooth movement: The biology and clinical implications. Kaohsiung J. Med. Sci..

[16] Bilgili E., Üçok C.Ö. (2023). Evaluating trabecular microstructure of mandible and axis in osteoporosis, diabetes and chronic kidney disease using cone beam computed tomography. Oral. Radiol..

[17] Vogel J.O., Freire C.H., Munhoz L., Andrade B.A.B., Tenório J.R. (2024). Mandibular bone imaging assessment in chronic kidney disease: A systematic review and meta-analysis. Oral Surg. Oral Med. Oral Pathol. Oral Radiol..

[18] Güneş G., Doğruer Ünal N., Eskandari G., Kiykim A., Bölgen Çimen Ö., Temel G., Çimen M.B.Y. (2018). Determination of NF-κB and RANKL levels in peripheral blood osteoclast precursor cells in chronic kidney disease patients. Int. Urol. Nephrol..

[19] Lu H., Wu H., Yang Y., Feng X., Ma X., Xie Y., Xie D., Wang W., Lo E.C.M., Ye W. (2022). Relationship between chronic periodontitis and inflammatory cytokines in patients undergoing maintenance hemodialysis. Clin. Oral Investig..

[20] Räisänen I.T., Aji N.R.A.S., Sakellari D., Grigoriadis A., Rantala I., Pätilä T., Heikkilä P., Gupta S., Sorsa T. (2023). Active Matrix Metalloproteinase-8 (aMMP-8) Versus Total MMP-8 in Periodontal and Peri-Implant Disease Point-of-Care Diagnostics. Biomedicines.

[21] Teles F.R.F., Chandrasekaran G., Martin L., Patel M., Kallan M.J., Furquim C., Hamza T., Cucchiara A.J., Kantarci A., Urquhart O. (2024). Salivary and serum inflammatory biomarkers during periodontitis progression and after treatment. J. Clin. Periodontol..

[22] Borujeni S.I., Mayer M., Eickholz P. (2015). Activated matrix metalloproteinase-8 in saliva as diagnostic test for periodontal disease? A case-control study. Med. Microbiol. Immunol..

[23] Sorsa T., Alassiri S., Grigoriadis A., Räisänen I.T., Pärnänen P., Nwhator S.O., Gieselmann D.R., Sakellari D. (2020). Active MMP-8 (aMMP-8) as a grading and staging biomarker in the periodontitis classification. Diagnostics.

[24] Chaparro A., Realini O., Hernández M., Albers D., Weber L., Ramírez V., Param F., Kusanovic J.P., Sorsa T., Rice G.E. (2021). Early pregnancy levels of gingival crevicular fluid matrix metalloproteinases -8 and -9 are associated with the severity of periodontitis and the development of gestational diabetes mellitus. J. Periodontol..

[25] Kloukos D., Mavrogonatou E., Kletsas D., Makras P., Koukos G., Stavropoulos A., Katsaros C. (2022). Bone turnover markers in gingival crevicular fluid and blood serum of patients with fixed orthodontic appliances. Eur. J. Orthod..

[26] Cavalla F., Letra A., Silva R.M., Garlet G.P. (2021). Determinants of Periodontal/Periapical Lesion Stability and Progression. J. Dent. Res..

[27] Massy Z., Drueke T. (2017). Adynamic bone disease is a predominant bone pattern in early stages of chronic kidney disease. J. Nephrol..

[28] Teodorescu A.C., Martu I., Teslaru S., Kappenberg-Nitescu D.C., Goriuc A., Luchian I., Martu M.A., Solomon S.M., Mârțu S. (2019). Assessment of Salivary Levels of RANKL and OPG in Aggressive versus Chronic Periodontitis. J. Immunol. Res..

[29] Ubuzima P., Nshimiyimana E., Mukeshimana C., Mazimpaka P., Mugabo E., Mbyayingabo D., Mohamed A.S., Habumugisha J. (2024). Exploring biological mechanisms in orthodontic tooth movement: Bridging the gap between basic research experiments and clinical applications—A comprehensive review. Ann. Anat..

[30] Lahdentausta L.S.J., Paju S., Mäntylä P., Buhlin K., Tervahartiala T., Pietiäinen M., Alfthan H., Nieminen M.S., Sinisalo J., Sorsa T. (2018). Saliva and serum biomarkers in periodontitis and coronary artery disease. J. Clin. Periodontol..

[31] Yamada S., Tanaka S., Arase H., Hiyamuta H., Yoshizumi E., Tokumoto M., Nakano T., Tsuruya K., Kitazono T. (2021). Associations Between Surrogates of Skeletal Muscle Mass and History of Bone Fracture in Patients with Chronic Kidney Disease: The Fukuoka Kidney disease Registry (FKR) Study. Calcif. Tissue Int..

[32] Silva T.M.C., Alves L.A.C., Garrido D., Watanabe A., Mendes F.M., Ciamponi A.L. (2019). Health and oral health-related quality of life of children and adolescents with chronic kidney disease: A cross-sectional study. Qual. Life Res..

[33] Ong Z.H., Ng C.H., Tok P.L., Kiew M.J.X., Huso Y., Shorey S., Ng Y.P.M. (2021). Sources of Distress Experienced by Parents of Children with Chronic Kidney Disease on Dialysis: A Qualitative Systematic Review. J. Pediatr. Nurs..

[34] Sezer B., Kaya R., Kodaman Dokumacıgil N., Sıddıkoğlu D., Güven S., Yıldız N., Alpay H., Kargül B. (2023). Assessment of the oral health status of children with chronic kidney disease. Pediatr. Nephrol..

[35] Garcia Santaella N., Premoli Maciel A., Simpione G., da Silva Santos P.-S. (2020). Halitosis, reduced salivary flow and the quality of life in pre-kidney transplantation patients. J. Clin. Exp. Dent..

[36] Liu W.C., Wu C.C., Lim P.S., Chien S.W., Hou Y.C., Zheng C.M., Shyu J.F., Lin Y.F., Lu K.C. (2018). Effect of uremic toxin-indoxyl sulfate on the skeletal system. Clin. Chim. Acta..

[37] Sieklucka B., Pawlak D., Domaniewski T., Hermanowicz J., Lipowicz P., Doroszko M., Pawlak K. (2021). Serum PTH, PTH1R/ATF4 pathway, and the sRANKL/OPG system in bone as a new link between bone growth, cross-sectional geometry, and strength in young rats with experimental chronic kidney disease. Cytokine.

[38] Zacarias J.M.V., de Alencar J.B., Tsuneto P.Y., de Souza V.H., Silva C.O., Visentainer J.E.L., Sell A.M. (2019). The Influence of TLR4, CD14, OPG, and RANKL Polymorphisms in Periodontitis: A Case-Control Study. Mediat. Inflamm..

